# Methods and background characteristics of the TOHNN study: a population-based study of oral health conditions in northern Norway

**DOI:** 10.3402/ijch.v75.30169

**Published:** 2016-02-19

**Authors:** Gro Eirin Holde, Nils Oscarson, Anders Tillberg, Peter Marstrander, Birgitta Jönsson

**Affiliations:** 1 The Public Dental Health Service Competence Centre of Northern Norway, Tromsø, Norway; 2 Department of Clinical Dentistry, Faculty of Health Sciences, UiT the Arctic University of Norway, Tromsø, Norway; 3 Public Dental Health Care Service, Tromsø County Council, Tromsø, Norway; 4 School of Education, Health and Social Studies, Dalarna University, Falun, Sweden

**Keywords:** epidemiology, survey, dental health, adults, prevalence

## Abstract

**Objectives:**

The aim of the Tromstannen – Oral Health in Northern Norway (TOHNN) study was to investigate oral health and dental-related diseases in an adult population. This article provides an overview of the background of the study and a description of the sample characteristics and methods employed in data collection.

**Study design:**

Cross-sectional population-based study including a questionnaire and clinical dental examination.

**Methods:**

A randomly selected sample of 2,909 individuals (20–79 years old) drawn from the population register was invited to participate in the study. The data were collected between October 2013 and November 2014 in Troms County in northern Norway. The questionnaire focused on oral health-related behaviours and attitudes, oral health-related quality of life, sense of coherence, dental anxiety and symptoms from the temporomandibular joint. The dental examinations, including radiographs, were conducted by 11 dental teams in 5 dental offices. The examination comprised of registration of dental caries, full mouth periodontal status, temporomandibular disorders, mucosal lesions and height and weight. The participants were grouped by age (20–34, 35–49, 50–64 and 65–79) and ethnicity (Norwegian, Sámi, other European and other world).

**Results:**

From the original sample of 2,909 individuals, 1,986 (68.3%) people participated, of whom 1,019 (51.3%) were women. The highest attendance rate was among women 20–34 years old (80.3%) and the lowest in the oldest age group of women (55.4%). There was no difference in response rate between rural and urban areas. There was a positive correlation between population size and household gross income (p < 0.001) and education level (p < 0.001). The majority of Sámi resided in smaller municipalities. In larger cities, most participants used private dental health care services, whereas, in rural areas, most participants used the public dental health care service.

**Conclusion:**

The TOHNN study has the potential to generate new knowledge on a wide range of oral health conditions beneficial to the population in Troms County. Due to the high participation rate, generalization both nationally and to the circumpolar area ought to be possible.

The 2 most common dental-related diseases are dental caries and periodontitis. Dental caries affects and causes destruction of the hard tissue of the teeth. According to a report from WHO (1), dental caries affects 60–90% of school-aged children and nearly 100% of most adult populations. The decayed, missing, filled teeth (DMFT) index among 35–44 year olds was high (>13.9) in 6 of the 8 circumpolar countries (Finland, Sweden, Norway, Iceland, Denmark and Canada) and moderate (9.0–13.9) in Russia and the United States (1). Periodontitis is an inflammatory disease affecting the periodontal tissue, where the host response induces tissue destruction that may lead to complete loss of teeth (2). Severe periodontitis affects 5–15% of the general population (3). Poor oral health can affect the general health and is related to chronic diseases (e.g. diabetes). Oral diseases like caries, periodontitis, tooth loss and oral mucosal lesions can lead to pain and problems with eating and chewing, influencing social functioning and quality of life. Treating oral diseases results in high costs for society, as oral disease is the fourth most expensive disease to treat in most industrialized countries (1,4,5). There are few epidemiological studies describing oral health status of adults in Norway (6–10), and a description of oral health conditions in the population of the northern and arctic region is lacking. The 2 main epidemiological studies from Norway are the Trøndelag study (6–8) and the Oslo studies (9,10). The Trøndelag study was a cohort study describing dental health in terms of the presence and consequences of dental caries and used the DMFT index, whereas the Oslo study was restricted to a targeted age group (35-year olds) and described the prevalence of caries and periodontitis. Both studies report dental health improvement during the study period. Other smaller studies targeted particular groups within the population. Oral conditions among the elderly have been investigated by Rise and Heløe (11), Henriksen (12) and Henriksen et al. (13), and satisfaction with oral health in 65-year olds in Sweden and Norway has been assessed by Ekbäck et al. (14). For the general Norwegian adult population, a study from the 1970s (15) describes health data collected from 1 dental clinic and concludes the number of remaining teeth decreased with increasing age and decreasing income and/or social class. Furthermore, people with a high socio-economic status have less caries, better oral hygiene and periodontal conditions.

Some published studies assess adults’ dental health in Norway; however, epidemiological studies covering most of the oral health conditions and within all age groups of the adult population are lacking. The aim of the Tromstannen – Oral Health in Northern Norway (TOHNN) study was to investigate oral health and dental-related diseases in an adult population in the northern region of Norway. Due to different demographic and socio-economic characteristics and dental healthcare utility in the region, the overall study hypothesis was that there might be differences in oral health status between populations in rural areas and in urban areas (7,16). Furthermore, there might also be differences in oral health and related variables between the northern and southern regions due to different living condition in the arctic region. This article provides an overview of the background of the study and a description of the sample characteristics and methods employed in data collection.

## Methods

This cross-sectional, population-based study included a structured questionnaire and a clinical examination. All data were collected between October 2013 and November 2014, in Troms County in northern Norway. The regional committees for medical and health research ethics of the University of Tromsø, Norway, approved the study (2013/348/REK Nord), and all participants provided both oral and written informed consent before the start of the study.

### Troms County

**Fig. 1:**
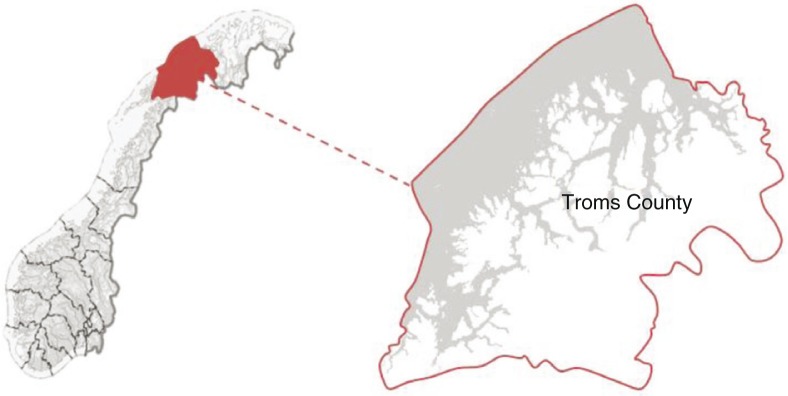
Troms County in Norway © Kartverket www.kartverket.no

Troms County is located in the northern part of Norway, above latitude 68°N (Fig. 1), and has about 163,500 inhabitants (17). The majority of the population resides in the cities of Tromsø (69.4°N: 70,358 inhabitants) and Harstad (68.5°N: 24,291 inhabitants). Tromsø is the administrative centre of the county and is a centre for education, commerce and transportation. Harstad is an important centre of commerce for the region and a trading centre for the southern part of the county. Tromsø city may be considered as a representative of northern European, urban population. The remaining inhabitants are spread throughout the rural areas of the county, and several inhabitants live on smaller islands, with time-consuming transportation to urban areas.

### Participants and study size

A randomly selected sample of 3,000 individuals (20–79 years old) drawn from the population register at Statistic Norway was invited to participate in the study. In this age group, 112,253 people were registered in Troms County in January 2013. A power calculation, with a 2-sided, 95% confidence interval and a width of 3%, indicated 1,537 individuals were required to be able to describe the prevalence of a disease (periodontitis/dental caries) occurring in approximately 10% of the population. The total sample was based on a 50% attendance rate experienced in other epidemiological studies in Norway, which reported an attendance rate from 29 to 64% (6,7,10,18). The approximation of a 10% occurrence, that is, for events of more severe periodontitis and dental caries, was based on data presented in other epidemiological studies (9,19–21). The reported frequency of advanced periodontitis in most countries appears to range from 8 to 13%.

To be able to detect possible differences between people living in rural areas and in an urban area (the city of Tromsø), the sample was stratified on 3 different areas in the county: Tromsø city (51,110 people: 46%), Southern Troms County (49,740 people: 44%) and northern Troms County (11,403 people: 10%). This resulted in 1,380 people from Tromsø city, 1,320 people from Southern Troms County and 300 people from northern Troms County being invited to participate. The participants were grouped by age (20–34, 35–49, 50–64 and 65–79) and ethnicity (Norwegian, Sámi, other European and other world).

### Invitation procedure

The study subjects were invited to participate by mail. The invitation letter included information written in Norwegian regarding the purpose of the study, examination procedures, the actions taken to ensure confidentiality and stated they would be contacted by telephone. Contact information was also provided for any questions regarding the study. A couple of weeks after the invitation was mailed, 6 trained callers that were familiar with the study and the details of the examination called the participants by telephone to confirm or decline participation. The callers all spoke Norwegian and used neutral language like the example below:

Hi, my name is […] and I am calling from […]. Have you received an invitation to participate in a study regarding oral health? Have you had the time to read the information? Would you like me to tell you about the study? Have you considered participation?

If they did not want to participate, they were asked if they wanted to give the reasons for this. Participants that were indecisive about participation were asked if they wanted to be contacted later. In cases where participants could not be reached by telephone, an additional letter was sent. For those who did not respond and could not be contacted by phone, an additional letter was sent out with simplified information.

Those who agreed to participate received a questionnaire and forms for written consent and medical history to be completed prior to the dental examination. The information about the study was repeated orally at the time of the examination. The examination was free of charge and travelling expenses were reimbursed. In a few cases, travelling expenses were prepaid. After the clinical examination, the participants received a gift card with a value of NOK150 (€18.12). The participants were also entered into a lottery for 2 tablet computers (iPad) and 20 power dental brushes. For those who declined participation, the reason for not attending was registered.

### Theoretical framework

**Fig. 2:**
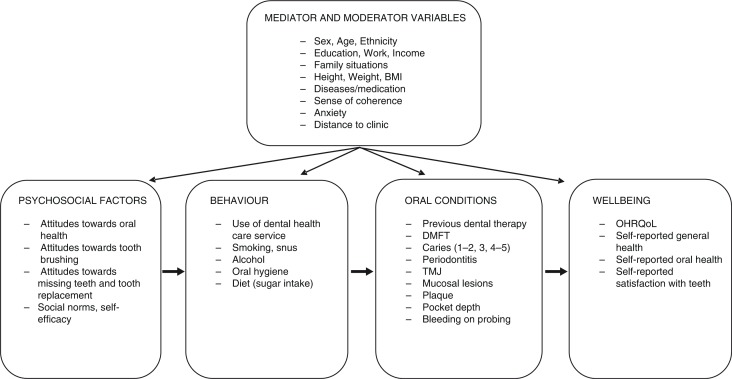
Theoretical model of variables measured in the TOHNN study.

During the planning process of the study, a theoretical framework was created to ensure the different aspects of oral health were assessed and measured in the questionnaire and clinical examination (Fig. 2). The general hypothesis was that the most common oral and dental disorders would be affected by an individual's behaviour. As different psychosocial factors influence a person's behaviour, some psychosocial assessments were included. It was hypothesised that different oral and dental conditions would influence a person's well-being. Therefore, patient-reported outcomes were included to capture the population's own view of oral health and to assess how different dental disorders can affect the quality of life. An additional hypothesis was that different moderating and mediating variables would influence the relationship between the different variables in the model.

### Questionnaire

The questionnaire was written in Norwegian and was tested on personnel without scientific or dental background. The participants completed the self-reported questionnaire prior to the examination. The 15-page questionnaire was developed by the authors comprising mostly formerly used questions from comparable studies and some new questions about general health and use of medication based on the HUNT study. Briefly described, the questions included background characteristics; socio-economics; dental healthcare services; oral hygiene-related behaviours (tooth brushing, interdental cleaning, etc.); attitudes toward oral health, previously used in a Norwegian population (18); dietary habits (22,23); subjective norms; normative beliefs; perceived behavioural beliefs/self-efficacy (24,25); dental anxiety scale (26,27); sense of coherence (28–30); oral health-related quality of life (31,32); symptoms of pain, such as headache or symptoms from the temporomandibular joint (33) and attitudes towards and perceived treatment needs (34,35) (Table I).

**Table I:** List of self-reported variables collected in the questionnaire and clinical measures registered

Measurement	Description
Q1–6 Background characteristics	Age, gender and ethnicity (18)
Q7–10 Socio-economics	Education, employment, income and marital status (18)
Q11–18a Use of dental health care services	Frequency of dental visits, public or private dental services and influence of costs on dental treatment (18)
Q18b Perceived treatment needs	Subjective need of dental treatment (34)
Q19–25 General health	Prescription medicine, health conditions and tobacco habits
Q26–28 Food-frequency questionnaire	Frequency of sugary foods and drinks, including alcohol (22,23)
Q29–32 Oral hygiene behaviour	Frequency of brushing, oral hygiene aids and fluoride (18)
Q33 Subjective norms, normative beliefs and self-efficacy	Questions about brushing behaviour developed from Theory of Planned Behaviour (24,25)
Q34 Sense of coherence (SOC)	Questions about SOC translated into Norwegian, according to authorised procedures (29). The SOC questionnaire is based on self-report. Several studies have found support for its validity and reliability (28,30).
Q35 Attitudes towards oral health	Importance of oral hygiene and oral health (18)
Q36 Oral health-related quality of life (OHIP-14)	Perception regarding discomfort and dysfunction caused by oral conditions (31,32)
Q37 Dental anxiety scale	Dental Anxiety Scale describing imagined dental situations (26,27)
Q38 Attitudes towards replacing	Importance of replacing teeth
Q39–42 Need and demand for prosthodontic treatment	Self-reported need and demand for prosthodontic treatment and opinions regarding dental implants (35)
Q43–49 Symptoms of pain	Pain or discomfort from mouth or face/jaw (33)
Height and weight	Calibrated height measurers and weights (KaWe PERSON-CHECK, ADE Class III approved scales, Electronic Floor scales M304044-02)
Previous dental therapy	Registration of restorative therapy (fillings, crown, etc.) and missing teeth
Dental caries	Registered according to diagnostic criteria (36)
Periodontal conditions	Parameters a–d (19,37,38)
a. Bleeding on probing	Periodontal probe North Carolina Probe–1, 2, 3, 4, 5, 6 ….15 mm, UNC15
b. Periodontal pocket depth	LM1100-EX (TECHNOMEDICS) on 6 surfaces of each tooth
c. Attachment loss	Radiographic bone loss measured to the nearest 10% (39)
d. Oral hygiene	Presence of plaque registered on 4 tooth surfaces (40)
Temporomandibular disorders	Palpation of temporomandibular joint, masticatory muscles, maximal mouth opening capacity by Helkimo index (41)
Occlusion	Number of supporting zones by Eichner index (42)
Mucosal lesions	Documented by clinical photos for further diagnosis
Treatment needed	Dentist's subjective evaluation

### Clinical and radiographical examinations

Clinical measures collected in the study were previous dental therapy, dental caries (36), periodontal conditions (19,37–39), oral hygiene (40), temporomandibular disorder (41), number of supporting zones (42) and height and weight (Table I). Intra and extra oral radiographs were taken (4 bitewing radiographs and 1 orthopantomogram). Each clinical and radiographic examination required between 45 and 90 minutes. The examinations were carried out by 11 dental teams in 5 dental offices located from north to south in the county. The offices were equipped with digital imaging and orthopantomogram machines, digital camera, a dental chair, an operation lamp, mouth mirror and examination probes. All clinical data were registered in a computerised protocol (Carestream-T4, Planmeca-OPUS) on a secured server.

**Table II:** Education level, household gross income, ethnicity and use of dental health service by municipality size

		Municipality size								
		
		<20,000		20,000–50,000		>50,000		Total		
						
		n	%	n	%	n	%	n	%	*p*
Education level	Secondary school	166	21.1	32	10.3	119	13.6	317	16.1	
	High school	374	47.6	144	46.5	332	38.1	850	43.2	<0.001
	University	245	31.2	134	43.2	421	48.3	800	40.7	
Total		785		310		872		1,967		
										
Household gross income (NOK)	<300,000	128	16.8	38	12.5	126	15.0	292	15.3	
	301,000–600,000	300	39.4	105	34.4	252	29.9	657	34.4	<0.001
	601,000–900,000	224	29.4	96	31.5	266	31.6	586	30.7	
	>900,000	109	14.3	66	21.6	198	23.5	373	19.6	
Total		761		305		842		1,908		
										
Ethnicity	Norwegian	772	97.2	307	98.4	850	96.9	1,929	97.3	
	Sámi	14	1.8	2	0.6	10	1.1	26	1.3	0.059
	Europe	6	0.8	–	–	4	0.5	10	0.5	
	Other	2	0.3	3	1.0	13	1.5	18	0.9	
Total		794		312		877		1,983		
										
Dental health service	Public	280	36.4	44	14.3	178	20.8	502	26.0	
	Private	339	44.0	233	75.9	544	63.6	1,116	57.8	<0.001
	Public and private	145	18.8	28	9.1	91	10.6	264	13.7	
	University dental clinic	6	0.8	2	0.7	42	4.9	50	2.6	
Total		770		307		855		1,932		

### Examination reliability

To improve and secure the inter-examiner reliability, different precautions were taken during the study period. Prior to study start, all examiners were trained and calibrated regarding the diagnostic criteria and examination procedures for each field compared with a golden standard (1 of the authors/examiner N.O). In addition, each examiner received a diagnostic manual in which all measurements and the procedures for diagnostics were described. To increase consistency of the registration of dental caries and periodontal measurements, 2 calibration tests for dental caries and 1 for probing pocket depth were conducted during the study period. For dental caries, a set of bitewing radiographs were examined by all examiners, and congruency towards the golden standard was evaluated. For the measurement of probing pocket depth, 6 surfaces around 6 teeth were measured, and congruency towards the golden standard was compared to the nearest millimetre. The inter-examiner agreement for caries registration and periodontal pocket measurement was assessed between each of 10 examiners and 1 of the examiners as a golden standard using per cent agreement and Cohen's kappa (κ). For caries registration, inter-examiner agreement was assessed in 2 separate cases with a 3-month interval. In the first case, per cent agreement ranged from 75 to 100%, and the median κ value was 0.73 (quartile deviation 0.5–0.85). In the second case, per cent agreement ranged from 81.3 to 91.7%, and the median κ value was 0.77 (quartile deviation 0.74–0.79). For periodontal pocket measurement, inter-examiner agreement was measured once at the start of the study, where the per cent agreement ranged from 77.8 to 100% and the median κ value was 0.7 (quartile deviation 0.66–0.78).

### Statistical analyses

For data analysis, the study population was divided into 3 groups based on the number of residents in each municipality in the county: Group 1<20,000; Group 2 20,000–50,000; and Group 3>50,000 inhabitants. Chi-square test was used to detect any differences between municipality size and attendance rate and demographic/socio-economic characteristics. All statistical analyses were performed with IBM^®^ SPSS^®^ statistics 22.

## Results

**Fig. 3:**
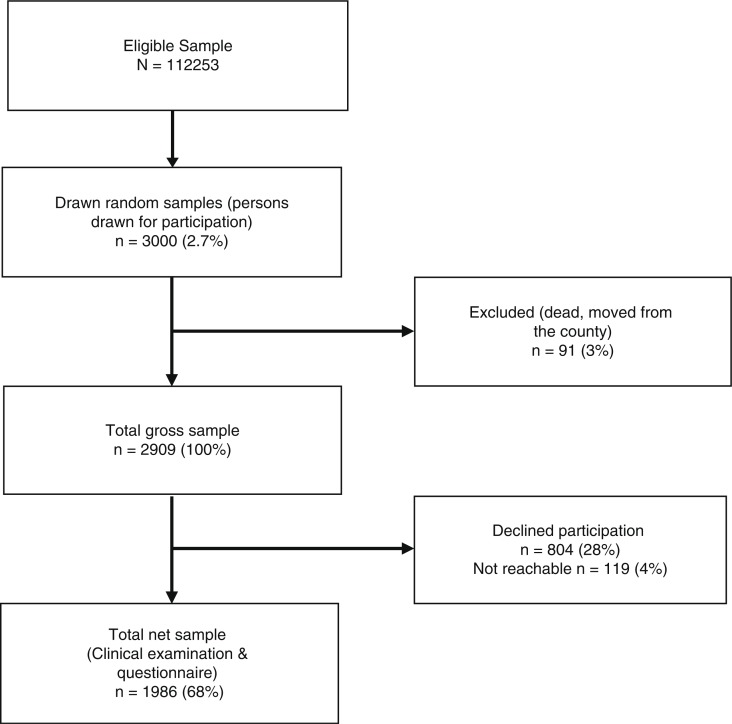
Flowchart of participants in the TOHNN study.

Originally, 3,000 people in Troms County were drawn from the population register. Excluding those who had moved from the county or had died (n = 91), the study population reduced to 2,909 individuals (Fig. 3). The number of individuals declining participation, or who could not be reached by phone or mail, was 922, which resulted in 1,986 participants who attended the examination and completed the questionnaire. This gave a response rate of 68.3%. The participants consisted of 967 (48.7%) men and 1,019 (51.3%) women, with a mean age of 48.0 years (SD 15.6). Stratified by age, the highest attendance rate was among women in the youngest age group, where 80.3% (n=269) attended the clinical examination. The lowest response was among women in the oldest age group (55.4%). For men and women in total, the age group 20–34 years had the highest response of 72.9%, whereas the age group 65–79 years had the lowest participation (57.3%).

Attendance rate varied between rural and urban areas, from 67.4% in the most sparsely populated areas to 69.4% in the largest city; however, no significant differences were found between rural and urban areas.

For self-reported ethnicity, the largest city had the highest proportions of participants with ethnicity other than Norwegian (Table II). The majority of Sámi resided in the smaller municipalities. There was a correlation between rural and urban areas, annual household gross income and education levels, where reported income and education level increased with population size. For the larger cities, most participants reported use of private dental healthcare services, whereas in rural areas a higher proportion used the public dental healthcare service.

## Discussion

The methods and background characteristics of the TOHNN study has been described. The high participation rate, especially among young women, was one of the main observations.

One reason for the high participation could be that the invitation was followed up by a personal phone call made by dental healthcare personnel with detailed knowledge about the study. The offer of compensation for travelling expenses and flexible times for the clinical examination probably facilitated recruitment. Furthermore, for participants with travelling difficulties, specific arrangements were made (i.e. taxi) to facilitate participation. The study was marketed in the media (radio, local newspaper and Facebook) to increase awareness of and interest in the study. Preliminarily, it was assumed there would be a lower response from rural areas due to longer travelling distances. Consequently, this was taken into consideration at an early stage, and effort was made to facilitate those with a long or difficult travel distance. As reported in the results, there was no significant difference between rural and urban areas regarding participation rate.

Reported income and education level increased with population size and supported the findings of Norheim (15) when describing health data in northern Norway. The use of public dental healthcare services was more common among those who lived in the smallest municipalities (<20,000 inhabitants), and 1 explanation could be a lower provision of private dental healthcare services in rural areas.

There are some limitations to mention: Since the study is cross-sectional, no causal relationship can be established. The oldest age group had a low response rate; however, similar attendance rates have been reported for seniors (65 years and older) (12,14,21,43). In this group, the most common reasons for not attending were health problems and no subjective need for dental health care. This can cause an under- or overestimation of oral health problems among the oldest age group and must be considered in future analysis of the material.

As the study subjects lived in a widespread geographical area in the county,11 dentists working at 1 of the 5 dental offices, located from north to south, were involved in data collection. This presented a possible variation in registration and diagnosis of disease. To ensure accuracy and consistency, all examination teams were trained and evaluated to a golden standard prior to the survey. In addition, inter-examiner agreement for registration of caries and periodontal probing depth was assessed during the course of the study and considered acceptable.

An ethical consideration is that the participants were contacted by a personal telephone call. By calling the participants by phone, they may have felt pressure to participate. On the other hand, we experienced that by calling, the potential participants would get the chance to ask questions and receive information about the study to enable an informed consent. Furthermore, when calling, those who had not received the invitation, misplaced it or confused it with advertisement would get a chance to participate and thereby preventing exclusion of these groups. Decision not to participate was respected and there was no pressure of individuals to participate. The recruitment procedure was approved by The Regional Ethics Committee (REC).

A few of the participants did not speak Norwegian, and the questionnaire had to be orally translated to English at the time of the examination. This could have lead to misinterpretations of the questions. If the participant was unsure about the meaning of the question, the question was excluded from the questionnaire.

Despite some of the limitations, the TOHNN study has several strengths: the study covers the total population from 20 to 79 years within Troms County, including both urban and rural areas. Future research based on the TOHNN database will generate new knowledge on a wide range of oral health conditions in the northern region of Norway. This knowledge will be of benefit locally for the adult population in Troms County and to The Public Dental Health Services in terms of planning interventions and future needs for oral health care. This new knowledge can also generate new hypotheses for future research projects. Due to the high participation rate, generalization both nationally and to the circumpolar area ought to be possible.

## Conclusions

In conclusion, the TOHNN study has the potential to generate new knowledge on a wide range of oral health conditions beneficial to the population in Troms County. Due to the high participation rate, generalization both nationally and to the circumpolar area ought to be possible.
